# SNP Typing Using Multiplex Real-Time PCR Assay for Species Identification of Forensically Important Blowflies and Fleshflies Collected in South Korea (Diptera: Calliphoridae and Sarcophagidae)

**DOI:** 10.1155/2019/6762517

**Published:** 2019-03-11

**Authors:** Hari Jang, Sang Eon Shin, Kwang soo Ko, Seong Hwan Park

**Affiliations:** Department of Legal Medicine, Korea University College of Medicine, 73, Inchon-ro, Seongbuk-gu, Seoul 02841, Republic of Korea

## Abstract

Medicolegal entomology—a subfield of forensic entomology—is mainly used in medicolegal investigations to estimate the postmortem interval (PMI). The minimum PMI of a corpse invaded by necrophagous immature insects can be estimated because the PMI is near to or earlier than the oviposition time of the larvae that hatched and fed on the corpse. As the growth speeds of larvae differ depending on temperature and species, species-specific growth data are used to estimate the minimum PMI. While morphological identification of adult necrophagous flies can be done by a well-trained entomologist, identification of larvae is relatively difficult. Larvae can only be identified up to the family level and developmental stage by observing the posterior spiracles. For these reasons, the molecular biology method of DNA barcoding has been developed. DNA barcoding that targets the mitochondrial cytochrome c oxidase subunit I (COI) gene is commonly used. COI sequences are currently acquired using polymerase chain reaction (PCR) and Sanger sequencing, which are too time-consuming and complex for practical use in medicolegal investigations. To compensate for these limitations and facilitate the use of entomology for medicolegal investigation, we designed a multiplex real-time PCR system to identify nineteen forensically important species of Calliphoridae and Sarcophagidae flies collected in South Korea. In contrast to the Sanger nucleotide sequencing process, this technology only requires a one-step real-time PCR with melt curve analysis of amplicons generated by primers targeting species-specific single nucleotide polymorphisms (SNPs). Multiplex real-time PCR was performed for twelve species of Calliphoridae (four reactions) and for seven species of Sarcophagidae (three reactions). This assay is expected to make it easier and faster for investigating authorities to identify major species of necrophagous flies at beginning of investigation and to increase the utilization of entomological evidence in forensic investigations.

## 1. Introduction

The minimum postmortem interval (PMI) of a dead body can be estimated using larvae hatched on the body because the minimum PMI is near to or earlier than the oviposition time [1]. As the growth speeds of larvae differ depending on temperature and species, species-specific growth data are needed to estimate the oviposit point [2]. To identify the species of necrophagous flies found on a dead body, morphological identification and molecular identification are performed [3]. Morphological identification of adult necrophagous flies can be done by a well-trained entomologist; identification of larvae is more difficult and complicated [1]. Larvae can be identified up to the family level and developmental stage (1st instar, 2nd instar, 3rd instar, and postfeeding) by observing their posterior spiracles [4] or by allowing maturation to occur to enable using adult identification keys. To address these disadvantages of morphological methods, molecular biological methods such as DNA barcoding have been developed. Previous DNA sequences studied include cytochrome c oxidase subunit I (COI) [5, 6], partial genomic sequences of the bicoid (bcd) gene [7], internal transcribed spacer (ITS2) [8], abdominal-B homeobox, and 16S rDNA [9]. Of these, DNA barcoding that targets COI is most commonly used. DNA sequences are obtained by Sanger sequencing; however, this method is time-consuming and complex, limiting the use of medicolegal entomology by criminal investigation authorities. Moreover, to identify species with acquired nucleotide sequences, knowledge of molecular phylogeny is required.

To compensate for these limitations, we designed a rapid and simple assay using multiplex real-time polymerase chain reaction (PCR) to identify 12 species of forensically important Calliphoridae and seven species of Sarcophagidae flies commonly found in South Korea [10–12]. This assay does not require obtaining and analyzing COI sequences of each sample by Sanger sequencing. Rather, it is based on analyzing melt curves of amplicons acquired from real-time PCR reactions using a double-stranded DNA-specific fluorescent dye, such as SYBR Green [13]. Melt curve shapes and melting temperatures of amplicons vary with GC/AT ratio, amplicon length, and sequence. These factors can be used to differentiate amplicons by gaps in melting temperature [14]. Duplex and multiplex real-time PCR using SYBR Green to analyze melt curves have been studied for use in detecting bacteria, viruses, and common domestic species in the domains of food safety, pathological diagnosis, and medicine and food authentication [15–17]. To acquire the melting temperature of each amplicon, we performed singleplex real-time PCR using species-specific primers that included two to five single nucleotide polymorphisms (SNPs); then, we combined two or three primer sets with gaps in melting temperature for multiplex real-time PCR.

## 2. Materials and Methods

### 2.1. Sample Collection

Adult flies and larvae belonging to twelve Calliphoridae and seven Sarcophagidae species were collected from Gyeonggi Province, Busan, and Seoul using traps or during autopsy. The Calliphoridae species we used were* Aldrichina grahami* (Aldrich, 1930),* Calliphora lata* (Coquillett, 1898),* Calliphora vicina* (Robineau-Desvoidy, 1830),* Calliphora vomitoria* (Linnaeus, 1758),* Chrysomya megacephala* (Fabricius, 1794),* Chrysomya pinguis* (Walker, 1858),* Lucilia caesar* (Linnaeus, 1758),* Lucilia illustris* (Meigen, 1826),* Lucilia sericata* (Meigen, 1826),* Phormia regina* (Meigen, 1826), and* Triceratopyga calliphoroides* (Rohdendorf, 1931). The Sarcophagidae species were* Sarcophaga albiceps* (Meigen, 1826),* Sarcophaga crassipalpis* (Macquart, 1839),* Sarcophaga dux* (Thomson, 1869),* Sarcophaga haemorrhoidalis* (Bottcher, 1913),* Sarcophaga melanura* (Meigen, 1826),* Sarcophaga peregrina* (Robineau-Desvoidy, 1830), and* Sarcophaga similis* (Meade, 1876). The samples were identified morphologically and preserved in 70% ethanol at −20°C.

### 2.2. DNA Extraction and COI Barcoding

Genomic DNA was extracted using Exgene™ Tissue SV mini Kit (GeneAll; Seoul, Korea) according to the manufacturer's protocol. Samples were preserved in 70% ethanol after extraction. Nucleic acids were eluted in sterilized distilled water. DNA concentrations were determined using a NanoDrop 2000c Spectrophotometer (Thermo Fisher Scientific; Wilmington, Delaware, USA). Aliquoted DNA was diluted to 1 ng/*µ*L for experiments and stored at -20°C. To identify the samples used in this study, universal primers were used for Sanger sequencing targeting COI regions [10]. Universal primers were synthesized using a MerMade 192 (BioAutomation; USA) by Macrogen Inc. (Macrogen; Daejeon, Korea). Fragments from Sanger sequencing were assembled using ChromasPro 2.1.5. (Technelysium Pty Ltd; South Brisbane, Australia) to obtain full COI region sequences.

### 2.3. Design of Species-Specific Primers Using SNPs

Primers were designed to be species-specific. To obtain the consensus sequences of each species, reference sequences from the National Center for Biotechnology Information (NCBI) GenBank (Table 1.) were aligned using Molecular Evolutionary Genetics Analysis (MEGA) 7.0.25 software [18]. Primers were designed based on COI and tRNA^Leu^ regions. Each primer set included two to five SNPs on its 3′ end. Primer 3 Web 0.4.0 [19] was used to design primers. Every primer was designed with a melting temperature of 52–64°C, GC-content of 30–60%, and amplicon melting temperature of 73–79°C. Each primer set was designed to target one or two species (Tables 2 and 3). Every primer was synthesized using a MerMade 192 (BioAutomation; USA) by Macrogen Inc. (Macrogen; Daejeon, Korea).

### 2.4. SYBR Green Real-Time PCR

This study was performed using a StepOnePlus™ Real-Time PCR System instrument (Applied Biosystems; Foster City, CA, USA). PCR was performed in duplicate or triplicate in a total reaction volume of 20 *µ*L: TOPreal™ qPCR 2X PreMIX (SYBR Green with high ROX, Enzynomics; Daejeon, Korea), 0.2 *μ*M of forward primer, 0.2 *μ*M of reverse primer, sterile water, and 1 ng of DNA. The amplification protocol consisted of initial denaturation at 95°C for 10 min, then 23 cycles of denaturation at 95°C for 10 s, annealing at 62°C for 15 s, and elongation at 72°C for 20 s. After the cycling stage, the melt curve stage was performed from 65°C to 85°C, with gradual temperature increases in increments of 0.3°C to acquire the melting temperature of the amplicon. We performed singleplex real-time PCR to determine the melting temperature of PCR products amplified using species-specific primer sets. Each species sample was tested with species-specific primer sets with included nontemplate controls. Samples from Calliphoridae and Sarcophagidae species were processed separately.

Multiplex real-time PCR was performed with combinations of primers with sufficiently different temperatures between amplicons for successful species-specific amplification. Based on the melting temperature of each amplicon obtained in the preceding singleplex reaction, a combination for the multiplex reaction was made. For each combination, two or three primer sets with amplicon melting temperature differences of about 2°C or more were used to allow identification of species by melting temperature. The combinations used in the assay were CC (combination of primers for Calliphoridae) and CS (combination of primers for Sarcophagidae). There were four combinations for CC (Table 4) and three for CS (Table 5). For normalization, the concentrations of each primer used in the multiplex reaction were optimized: CC1 consisted of 0.25 *μ*M of primer “mg,” 0.30 *μ*M of “il/ca,” and 0.35 *μ*M of “re.” CC2 consisted of 0.20 *μ*M of primer “la,” 0.20 *μ*M of “am,” and 0.15 *μ*M of “ca.” CC3 consisted of 0.15 *μ*M of primer “gr,” 0.30 *μ*M of “vi,” and 1.50 *μ*M of “vo.” CC4 consisted of 0.10 *μ*M of primer “pi,” 1.50 *μ*M of “se,” and 0.1 *μ*M of “cl.” CS1 consisted of 0.10 *μ*M each of primer “me” and “pg.” CS2 consisted of 0.15 *μ*M of primer “du” and 0.10 *μ*M of primer “si.” CS3 consisted of 1.20 *μ*M of primer “al,” 0.15 *μ*M of “cr,” and 0.10 *μ*M of “hm.”

## 3. Results and Discussion

### 3.1. Multiplex Real-Time PCR

**Figure 1:**
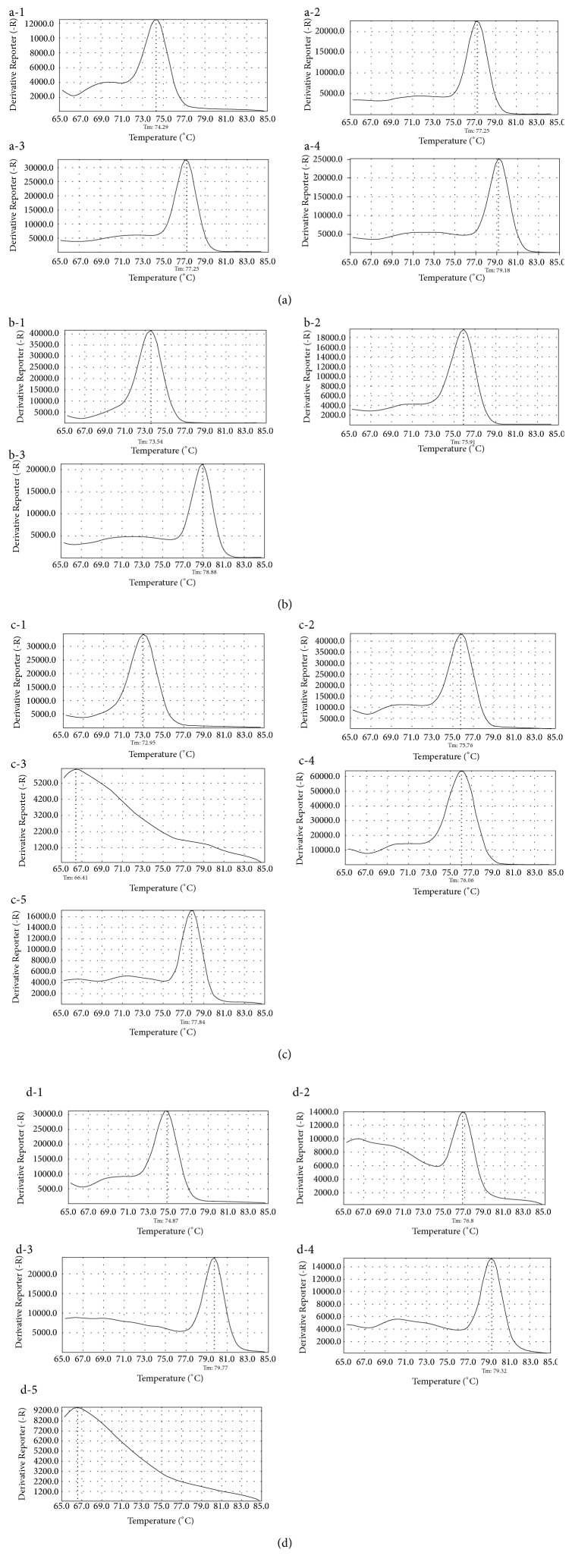
Melt curves of amplicons using primer combinations (Calliphoridae). (a)* CC1*: (1)* P. regina* (*T*_*m*_ = 74.29°C). (2)* L. illustris* (*T*_*m*_ = 77.25°C). (3)* L. caesar* (*T*_*m*_ = 77.25°C). (4)* C. megacephala *(*T*_*m*_ = 79.15°C). (b**)*** CC2*: (1)* L. ampullacea* (*T*_*m*_ = 73.54°C). (2)* C. lata* (*T*_*m*_ = 75.91°C). (3)* L. caesar* (*T*_*m*_ = 78.88°C). (c)* CC3*: (1)* C. vicina *(*T*_*m*_ = 72.95°C). (2)* L. ampullacea *(*T*_*m*_ = 75.76°C). (3)* L. ampullacea* (*nonamplified*). (4)* A. grahami *(*T*_*m*_ = 76.06°C). (5)* C. vomitoria *(*T*_*m*_ = 74.87°C). (d)* CC4*: (1)* C. pinguis *(*T*_*m*_ = 74.87°C). (2)* L. sericata *(*T*_*m*_ = 76.80°C). (3)* T. calliphoroides* (*T*_*m*_ = 79.77°C). (4)* C. vicina *(*T*_*m*_ = 76.32°C). (5)* C. vicina *(*nonamplified*).

**Figure 2:**
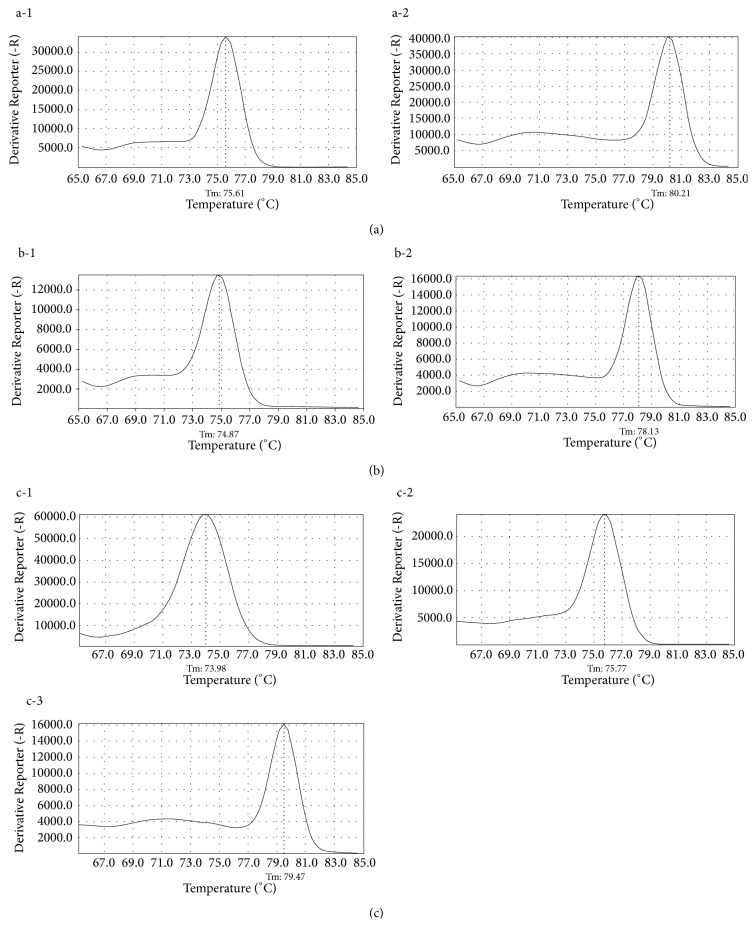
Melt curves of amplicons using primer combinations (Sarcophagidae). (a) Melt curves of amplicons generated by primer combination CS1. Number of figure and species amplified, from left to right, are as follows: a-1,* S. peregrina* (*T*_*m*_ = 75.61°C); a-2,* L. melanura* (*T*_*m*_ = 80.21°C). (b) Melt curves of amplicons generated by primer combination CS2. Number of figure and species amplified, from left to right, are as follows: b-1,* S. dux* (*T*_*m*_ = 74.87°C); b-2,* S. similis* (*T*_*m*_ = 78.13°C). (c) Melt curves of amplicons generated by primer combination CS3. Number of figure and species amplified, from left to right, are as follows: c-1,* S. haemorrhoidalis *(*T*_*m*_ = 73.98°C); c-2,* S. crassipalpis *(*T*_*m*_ = 75.77°C); c-3,* S. albiceps* (*T*_*m*_ = 79.47°C).

By analyzing the melt curves obtained from singleplex reactions, we confirmed the specificity of each primer; subsequently, we obtained the melting temperature of the amplicon when amplified using species-specific primers. The melting temperature of the amplicons was between 73°C and 79°C (Tables 6 and 7). All primers were designed to amplify only the target species, but there was amplification in nontarget species with variation in the primer-designed region, such as primer sets “gr” and “cl” (Table 2). In the case of “gr,” gDNA of* L. ampullacea* was amplified or not according to whether the 312th base of the COI sequence was C or T, respectively. In the case of “cl,” gDNA of* C. vicina* was amplified or not according to whether the 402nd base was C or T, respectively. In these cases, the ΔCt value—the difference between the Ct values—was used for precise identification. When amplified with primer “gr,” the ΔCt values were 3.0 and 4.1 for* A. grahami* and* L. ampullacea*, respectively. When amplified using primer “cl,” the ΔCt values for* T. calliphoroides* and* C. vicina* were 3.9 and 5.0, respectively. Primer “il/ca” is a primer that targets both* L. illustris* and* L. caesar* because of their high similarity. We performed multiplex PCR—using a primer combination based on the melting temperature obtained from the singleplex reaction—to confirm that only the target species was amplified in each of the combinations (Figures 1 and 2). There were some differences in melting temperature between multiplex and singleplex reactions. To normalize the value of the reporter fluorescence in the melt curve, we optimized the primer concentration. The value was normalized to be over 10,000 with 1 ng of DNA template, and the values <10,000 were considered invalid. From the results, the value of unexpected amplicons of* L. ampullacea* and* C. vicina* with SNPs in the primer-designed region measured under 10,000.

### 3.2. Assay Validation

#### 3.2.1. Detection of Amplicon

As the RT-PCR products were between 61 bp and 265 bp in length, NuSieve™ GTG™ Agarose (Lonza; Basel, Switzerland) was used as it finely resolves RT-PCR products ranging from 10 to 1000 bp. Amplicon samples in 6X Loading STAR (Dyne Bio; Seongnam, Korea) were run alongside a 25-bp DNA ladder (Promega; Madison, Wisconsin, USA) on a 3% gel (NuSieve™ 3:1 Agarose) in TAE buffer.

#### 3.2.2. Reproducibility Test

To validate the assay, blind and cross-reaction tests were performed. The blind test was performed with adult flies and larvae. Every sample used in the blind test was identified by molecular DNA barcoding using COI; the samples were tested randomly. Adult flies were identified morphologically, and larvae were identified to the family level using identification keys based on posterior spiracles. The blind test was performed with 81 Calliphoridae flies, 25 Sarcophagidae flies, and 10 Muscidae flies, including larvae and adults, to validate the applicability of the assay. The result showed a 100% concordance rate between Calliphoridae and Sarcophagidae (Table 8). We could not get the Ct value and invalid melting temperature in Muscidae with both primer combinations “CC” and “CS.” In testing any cross-reaction between Calliphoridae and Sarcophagidae with “CC” and “CS,” no valid amplification occurred. These results strongly support the applicability of this assay for Calliphoridae, Sarcophagidae, and Muscidae flies, all of which are commonly found in South Korea.

## 4. Conclusions

Medicolegal entomology is one way to estimate postmortem interval (PMI), but utilization is very low in Korea. This is not only because there are not many entomologists who can identify necrophagous flies morphologically, but also because of the disadvantages of existing molecular biology methods, which are time-consuming to use. Therefore, in order to improve the utilization of medicolegal entomology in criminal investigations, we aimed to design a quick and easy assay targeting the most common necrophagous flies found in Korea—Calliphoridae and Sarcophagidae.

Multiplex PCR was chosen to reduce consumption of reagents, gDNA, and time and to simplify the procedure to identify the species of flies. The singleplex method requires seven to twelve reactions to identify one sample, whereas the multiplex method can yield results with only three or four reactions. When a blind test was performed on the multiplex reaction, the melt curve was accurate and showed a specific melting temperature. This result proves that species identification of larvae collected from crime scenes can be done precisely in just four hours—even less time depending on the DNA extraction methods used. The multiplex real-time PCR itself takes only 70 min, while methods using Sanger sequencing take about 7 h. We also confirmed with a sensitivity test that the assay can be performed with 1 ng or more of gDNA. However, the authors recommend using 1 ng of gDNA in this assay to avoid unexpected amplification of untargeted species.

Because the experimenter needs only fly specimens, a DNA extraction kit, SYBR Green qPCR Mix, and a real-time PCR instrument, this assay has advantages in terms of cost and time compared to existing methods. The limitation of this assay is that species not considered in the design may or may not be amplified according to their similarity to the primers. Therefore, this assay is not suitable to apply to unreferenced species, but it can be redesigned with COI sequences of new species. Furthermore, this assay can be modified to identify necrophagous beetles such as Dermestidae and Silphidae.

In conclusion, this assay is expected to be useful for investigating authorities to identify species of necrophagous flies in initial investigation before obtaining full COI sequences of fly samples by Sanger's sequencing process and to increase the utilization of entomological evidence in forensic investigations.

## Figures and Tables

**Table 1:** List of reference sequences, accession numbers retrieved from the GenBank, and abbreviations of forensically important flies used in this study.

Family	Species Name	Accession Number	Abbreviation
Calliphoridae	*Aldrichina grahami*	EU880180-EU880182	Gr
	*Calliphora lata*	EU880183-EU880187	La
	*Calliphora vicina*	EU880188-EU880192	Vi
	*Calliphora vomitoria*	MG969488-MG969490, GQ223336	Vo
	*Chrysomya megacephala*	KM873618-KM873619	Mg
	*Chrysomya pinguis*	KM873620-KM873621	Pi
	*Lucilia ampullacea*	EU925394	Am
	*Lucilia caesar*	EU880193-EU880196	Ca
	*Lucilia illustris*	EU880197-EU880205	Il
	*Lucilia sericata*	EU880208-EU880212	Se
	*Phormia regina*	KX853042, AF295550	Re
	*Triceratopyga calliphoroides*	EU880176-EU880179	Cl

Sarcophagidae	*Sarcophaga albiceps*	JX861469-JX861473	Al
	*Sarcophaga crassipalpis*	KJ420597-KJ420599	Cr
	*Sarcophaga dux*	JX861474-JX8614745, EF405937-EF405939	Du
	*Sarcophaga haemorrhoidalis*	JX861406-JX861408, KM881633	Hm
	*Sarcophaga melanura*	JX861418-JX861419	Me
	*Sarcophaga peregrina*	JX861409-JX861412	Pg
	*Sarcophaga similis*	JX861476-JX861480	Si

**Table 2:** Information on species-specific primers (Calliphoridae).

Primer Set	Forward/Reverse	Sequence (5′ → 3′)	Amplicon Size (bp)	Target Species
gr*∗*	F	TGA CTT CTA CCC CCT GCA C	76	*A. graham* *(L. ampullacea)*
	R	CAG TTC ATC CTG TTC CAG CTC		

la	F	GTA GTT GCT CAT TTT CAT TAT GTG C	77	*C. lata*
	R	GGG TAT CAG TGT ACA AAT CCT GCT		

vi	F	CTG TCC CAA CAG GAA TTA AGA TTT TT	56	*C. vicina*
	R	TTG GGT ACC ATA AAG AGT TGC T		

vo	F	ATA CGA TCA ACA GGT ATT ACC TTC	163	*C. vomitoria*
	R	CTC CTG CTG GGT CAA AGA AT		

mg	F	CTT TGA TCC AGC AGG AGG AG	265	*Ch. megacephala*
	R	TGA AGT GAA ATA AGC TCG TGT GTC T		

pi	F	CGT AGA TAC TCG AGC TTA TTT CAC C	65	*Ch. pinguis*
	R	TCT TAA TTC CAG TTG GTA CAG CA		

am	F	TTC TAC AAT TGG ATC AAC AAT TTC TC	76	*L. ampullacea*
	R	AAC AAG ACT TTC TCA CAC AAT AAA GAA		

ca	F	GGA AAG AAG GAA ACA TTT GGG	207	*L. caesar*
	R	TTG AGT TCC GTA AAG AGT TGC T		

il/ca*∗∗*	F	GGA TTC GTT CAT TGA TAT CCT C	145	*L. caesar* *L. illustris*
	R	ATC GTC GTG GTA TTC CTG CT		

se	F	GAA ACA TTC GGT TCA TTA GGG ATG	113	*L. sericata*
	R	GCT CGT GTA TCA ACG TCT ATT CC		

re	F	TTG GAG GAT TTG GAA ATT GA	61	*P. regina*
	R	GGG AAA GCT ATA TCA GGT GCC		

cl*∗∗∗*	F	ATA TGG CTT TCC CTC GGA TAA AC	151	*T. calliphoroides* *(C. vicina)*
	R	GCT CCT CCA TGT GCG ATA TT		

*∗*: *L. ampullacea* may or may not be amplified with primer set “gr” due to variation.

*∗∗*: *L. illustris* and *L. caesar* are both amplified with primer set “il/ca.”

*∗∗∗*: *C. vicina* may or may not be amplified with primer set “cl” due to variation.

**Table 3:** Information on species-specific primers (Sarcophagidae).

Primer Set	Forward/Reverse	Sequence (5′ → 3′)	Amplicon Size (bp)	Target Species
al	F	TTT TTG GAG CTT GAG CAG GT	231	*S. albiceps*
	R	TTA TTC GAG GGA ATG CCA TG		

cr	F	ACC GGT ACT TGC AGG AGC TA	74	*S. crassipalpis*
	R	CTG CTG GGT CGA AAA AAG AG		

du	F	CTT TTA CCA CCA GCA TTA ACA TTA TTA	68	*S. dux*
	R	CAT CCT GTT CCA GCT CCA TT		

hm*∗*	F	CAA AAT ACA CCA CCA GCT GAA	65	*S. haemorrhoidalis* *= S. africa*
	R	tta gaT TAG AAG TTA GTT AGT AGC GGT		

me	F	GAG CCC CAG ATA TAG CTT TCC	208	*S. melanura*
	R	GAA GAG ATT CCA GCT AGA TGA AG		

pg	F	TAT GGC CGG ATT TGT TCA TT	135	*S. peregrina*
	R	GCT AAA CCT AAG AAG TGT TGG GGA		

si	F	TAC TTC ATT CTT TGA TCC TGC T	159	*S. similis*
	R	CCA AAT GTT TCC TTT TTA CCT GA		

*∗*: Primer set “hm” was designed based on the sequences of COI and tRNA^Leu^. Lowercase letters indicate sequences from tRNA^Leu^.

**Table 4:** Combinations of species-specific primers (Calliphoridae).

Combination Name	Primer Set	Sequence (3′ → 5′)	Optimized Concentration (*μ*M)	Target Species
CC1	mg	CTT TGA TCC AGC AGG AGG AG TGA AGT GAA ATA AGC TCG TGT GTC T	0.25	*Ch. megacephala*
	il/ca	GGA TTC GTT CAT TGA TAT CCT C ATC GTC GTG GTA TTC CTG CT	0.30	*L. illustris* *L. caesar*
	re	TTG GAG GAT TTG GAA ATT GA GGG AAA GCT ATA TCA GGT GCC	0.35	*P. regina*

CC2	la	GTA GTT GCT CAT TTT CAT TAT GTG C GGG TAT CAG TGT ACA AAT CCT GCT	0.20	*C. lata*
	am	TTC TAC AAT TGG ATC AAC AAT TTC TC AAC AAG ACT TTC TCA CAC AAT AAA GAA	0.20	*L. ampullacea*
	ca	GGA AAG AAG GAA ACA TTT GGG TTG AGT TCC GTA AAG AGT TGC T	0.15	*L. caesar*

CC3	gr	TGA CTT CTA CCC CCT GCA C CAG TTC ATC CTG TTC CAG CTC	0.15	*A. graham* *(L. ampullacea)*
	vi	CTG TCC CAA CAG GAA TTA AGA TTT TT TTG GGT ACC ATA AAG AGT TGC T	0.30	*C. vicina*
	vo	ATA CGA TCA ACA GGT ATT ACC TTC CTC CTG CTG GGT CAA AGA AT	1.50	*C. vomitoria*

CC4	pi	CGT AGA TAC TCG AGC TTA TTT CAC C TCT TAA TTC CAG TTG GTA CAG CA	0.10	*Ch. pinguis*
	se	GAA ACA TTC GGT TCA TTA GGG ATG GCT CGT GTA TCA ACG TCT ATT CC	1.50	*L. sericata*
	cl	ATA TGG CTT TCC CTC GGA TAA AC GCT CCT CCA TGT GCG ATA TT	0.10	*T. calliphoroides* *(C. vicina)*

**Table 5:** Combinations of species-specific primers (Sarcophagidae).

Combination Name	Primer Set	Sequence (3′ → 5′)	Optimized Concentration (*μ*M)	Target Species
CS1	me	GAG CCC CAG ATA TAG CTT TCC GAA GAG ATT CCA GCT AGA TGA AG	0.10	*S. melanura*
	pg	TAT GGC CGG ATT TGT TCA TT GCT AAA CCT AAG AAG TGT TGG GGA	0.10	*S. peregrina*

CS2	du	CTT TTA CCA CCA GCA TTA ACA TTA TTA CAT CCT GTT CCA GCT CCA TT	0.15	*S. dux*
	si	TAC TTC ATT CTT TGA TCC TGC T CCA AAT GTT TCC TTT TTA CCT GA	0.10	*S. similis*

CS3	al	TTT TTG GAG CTT GAG CAG GT TTA TTC GAG GGA ATG CCA TG	1.20	*S. albiceps*
	cr	ACC GGT ACT TGC AGG AGC TA CTG CTG GGT CGA AAA AAG AG	0.15	*S. crassipalpis*
	hm	CAA AAT ACA CCA CCA GCT GAA TTA GAT TAG AAG TTA GTT AGT AGC GGT	0.10	*S. haemorrhoidalis*

**Table 6:** Obtained melting temperatures (*T*_*m*_) of amplicons using primer combinations (Calliphoridae).

Combination Name	Amplified Species	*T*_*m*_ of Amplicon (°C)
CC1	*P. regina*	74.43±0.05
	*L. illustris*	76.95±0.37
	*L. caesar*	77.02±0.37
	*Ch. megacephala*	79.40±0.38

CC2	*L. ampullaceal*	73.74±0.80
	*C. lata*	75.84±0.52
	*L. caesar*	78.58±0.30

CC3	*C. vicina*	72.85±0.79
	*L. ampullaceal*	75.90±0.14 *or non-amplified*
	*A. graham*	75.92±0.29
	*C. vomitoria*	77.69±0.30

CC4	*Ch. pinguis*	74.86±0.42
	*L. sericata*	76.63±0.45
	*T. calliphoroides*	79.84±0.53
	*C. vicina*	79.40±0.08 *or non-amplified*

**Table 7:** Obtained melting temperatures (*T*_*m*_) of amplicons using primer combinations (Sarcophagidae).

Combination Name	Amplified Species	*T*_*m*_ of Amplicon (°C)
CS1	*S. peregrina*	75.15±0.46
	*S. melanura*	79.99±0.22

CS2	*S. dux*	74.20±0.95
	*S. similis*	77.77±0.52

CS3	*S. haemorrhoidalis*	74.05±0.21
	*S. crassipalpis*	75.75±0.31
	*S. albiceps*	79.19±0.59

**Table 8:** The results of the blind test using multiplex real-time PCR assay.

Family	Species Name	Typed/Total	Concordance Rate(%)
Calliphoridae	*Aldrichina graham*	1/1	100
	*Calliphora lata*	9/9	100
	*Calliphora vicina*	9/9	100
	*Calliphora vomitoria*	3/3	100
	*Chrysomya megacephala*	5/5	100
	*Chrysomya pinguis*	10/10	100
	*Lucilia ampullaceal*	4/4	100
	*Lucilia caesar*	10/10	100
	*Lucilia illustris*	5/5	100
	*Lucilia sericata*	10/10	100
	*Phormia regina*	10/10	100
	*Triceratopyga calliphoroides*	5/5	100

Sarcophagidae	*Sarcophaga albiceps*	1/1	100
	*Sarcophaga crassipalpis*	6/6	100
	*Sarcophaga dux*	10/10	100
	*Sarcophaga haemorrhoidalis*	1/1	100
	*Sarcophaga melanura*	2/2	100
	*Sarcophaga peregrina*	10/10	100
	*Sarcophaga similis*	5/5	100

## Data Availability

The data used to support the findings of this study are included within the article.
